# Effects of background statin therapy on glycemic response and cardiovascular events following initiation of insulin therapy in type 2 diabetes: a large UK cohort study

**DOI:** 10.1186/s12933-017-0587-6

**Published:** 2017-08-22

**Authors:** Uchenna Anyanwagu, Jil Mamza, Richard Donnelly, Iskandar Idris

**Affiliations:** Division of Medical Sciences & Graduate Entry Medicine, School of Medicine, University of Nottingham, Royal Derby Hospital, Uttoxeter Road, Derby, DE22 3DT UK

## Abstract

**Aim:**

Statins may increase the risk of new-onset diabetes and adversely affect glycaemic control, but their effects on the glycemic response and mortality outcomes following commencement of insulin therapy in patients with Type 2 Diabetes (T2D) are unclear.

**Methods:**

A retrospective cohort study was conducted in 12,725 insulin initiators with T2D using The Health Improvement Network (THIN) UK database. Changes in HbA1c at 6, 12, 24 and 36 months, and the 5-year risk of mortality and (3-point) major adverse cardiovascular events (MACE), were compared between prior users (n = 10,682) and non-users (n = 2043) of statin therapy who were newly commenced on insulin treatment. Cox proportional hazard models were used to estimate the hazard ratios of the different outcomes.

**Results:**

Mean age of the cohort was 58.7 ± 14.0 years (51% male) and mean baseline HbA1c was 8.7 ± 1.8%. A greater initial reduction in HbA1c was observed following insulin initiation in the non-users of statins compared with the users, which was significant in the short term (−0.34% vs −0.26% at 6 months; mean diff = −0.09%, p = 0.004) but not in the long term: −0.31% versus −0.35% at 3 years (mean diff = 0.05%, p = 0.344). CV events (3-point MACE) were 878 versus 217 in statin users versus non-users (20.7 vs 30.9 per 1000 person-years; adjusted Hazard Ratio (aHR) 1.36 (95% CI 1.15–1.62; p < 0.0001). In a subgroup analysis of individual statins, HbA1c was higher throughout the study duration with all statins relative to non-users of statin therapy (p < 0.05). The aHRs for 3-point MACE for atorvastatin, simvastatin, rosuvastatin and pravastatin were 0.82 (95% CI 0.68–0.98), 0.67 (0.55–0.82), 0.56 (0.39–0.81) and 0.78 (0.60–1.01), respectively.

**Conclusions:**

Following initiation of insulin therapy in patients with T2D in routine care, concurrent use of a statin was associated with less good glycaemic control in the short-term but a much lower risk of major adverse CV events.

**Electronic supplementary material:**

The online version of this article (doi:10.1186/s12933-017-0587-6) contains supplementary material, which is available to authorized users.

## Introduction

Evidence from randomized controlled trials shows that statin therapy reduces the risk of fatal and nonfatal cardiovascular (CV) events in patients with T2D [1–3]. Thus, clinical guidelines advocate their routine use in all patients with diabetes aged 40-75 years, and in younger patients with high CV risk [4, 5].

Various studies have shown that statins may also have modest adverse effects on glucose and insulin metabolism, e.g. increasing the risk of new-onset diabetes, especially in higher doses and with the more potent statins [6–9], but the relationship between statin use and glycaemic control in patients with established T2D is much less clear. Previous studies have reported an increase in HbA1c [10, 11] while others have reported either no effect or a reduction in glucose levels [12–14]. A meta-analysis of 9 randomized controlled trials (RCTs) involving 9696 participants reported that glycaemic control was adversely affected among those patients randomized to a statin compared with placebo, and that statin treatment also increased HbA1c in those patients with established T2D [15]. None of these studies, however, has specifically focused on T2D patients who are commencing insulin therapy, nor have they excluded the possibility of bias due to differential attrition rates and/or differential adjustment of other glucose-lowering therapies (GLTs) between the statin and control groups [16].

More information is certainly needed about the associations between statin use, glycaemic control and CV risk specifically in the insulin-treated T2D population, because these patients tend to have more complications, higher absolute CV risk and a longer duration of diabetes. Commencing insulin can also affect lipid levels and overall CV risk [17]. Thus, in routine practice some of the well-recognised clinical inertia may be due to uncertainty about the optimal timing and the overall risk–benefit balance of insulin initiation in these patients. For example, it was reported that primary prevention with statins was initiated in less than half of diabetic patients after a first MI, despite the presence of one or more markers of very high cardiovascular risk in nearly all [18]. Therefore, the aim of this large cohort study was to investigate the effects of background statin use on glycaemic control and CV outcomes following commencement of insulin therapy in patients with T2D in a routine primary care setting.

## Methods

### Study design

This was a retrospective cohort study using a UK primary care database.

### The Health Improvement Network (THIN) Database

THIN is a large UK electronic Primary Care database with longitudinal records obtained from approximately 587 General Practices. It contains details of over 12.4 million patients, of which 3.61 million were active as of January 2014. Trained doctors and specialist nurses systematically enter routine clinical information into this database. This includes specialist or Primary care consultations, diagnoses, laboratory results, prescriptions, referrals, hospital admissions, immunisations and important clinical measures such as body weight, height and body mass index (BMI). Information on the patients’ demography, lifestyle characteristics (e.g. alcohol use and smoking), socio-economic status (Townsend deprivation scores), ethnicity, religion and languages are also included. Several studies have validated the THIN database and shown it to be demographically representative of the wider UK population in terms of demography, disease prevalence and mortality [19]. Our own group and others have also used the THIN database to evaluate diabetes-related outcomes in routine clinical practice [20, 21].

Ethical approval for this study was obtained from the South-East Research Ethics Committee.

### Study participants

We obtained data on 12,725 people in the THIN database who had a diagnosis of T2D, were >18 years of age and who initiated insulin therapy between December 2006 and May 2014. Patients with type 1 or gestational diabetes, or other forms of diabetes, and those with no continuous regular prescriptions for insulin in their records were excluded. For analysis of our secondary objective, we excluded patients with a history of CV disease before or 180 days after initiating insulin.

### Follow-up and endpoints

We followed-up all insulin-initiators from the point they commenced statin treatment and compared them with those who did not commence statins until the first incident of any of the secondary outcomes: 3-point major adverse cardiovascular events (MACE: all-cause mortality or non-fatal myocardial infarction or stroke); loss to follow-up; discontinuation of insulin and/or statin therapy; or at the end of the 5-year follow-up period. Similarly, their baseline HbA1c, body weight and blood pressure were measured at baseline and at different time points after starting insulin (6, 12, 24 and 36 months).

The primary endpoints were glycaemic control (measured by change in HbA1c), changes in body weight, systolic blood pressure (SBP) and diastolic blood pressure (DBP) from baseline to 6, 12, 24 and 36 months after commencement of insulin treatment. These differences were computed and compared between the two treatment groups (statin users versus non-users). The secondary endpoint was the time to the risk of a 3-point MACE. These outcomes were identified by their appropriate Read Codes in the database.

### Baseline and endpoint characteristics

To adjust for the confounding effects some differences in baseline characteristics may have on the study outcomes, we extracted data on important clinical covariates. These included demographic variables such as age, gender, socioeconomic status, alcohol and smoking status; important clinical measures such as body weight, height, SBP and DBP; biochemical parameters, e.g. baseline HbA1c, serum creatinine, lipid-profile, use of other medications including other glucose-lowering therapies (GLTs); as well as comorbidity status, duration of diabetes treatment, and duration of insulin use. These were included in our univariate analysis models from which the significant covariates (those which had a significant association with both the exposure and outcomes) were added to the final Cox and linear regression models.

### Statistical analysis

We computed summary data for the mean, standard deviations and proportions of the baseline characteristics and used Pearson’s Chi squared test and *t* test to determine the differences between the treatment groups at baseline.

For the missing values in HbA1c, weight, SBP and DBP at baseline and all the study time points, we observed that a small proportion of values for these covariates were completely missing at random. These missing values were then computed using multiple imputations using the chained equation (MICE) model. Thereafter, linear regression models were used to compute the mean differences between baseline HbA1c, weight, SBP and DBP and the 6, 12, 24 and 36 month measurements respectively while adjusting for significant baseline covariates.

For the secondary endpoints of MACE, we used a Cox proportional hazard model to estimate the marginal and adjusted hazard ratios (HRs) with 95% confidence intervals, comparing the outcomes in the statin treated group to the non-user group. Furthermore, the crude and adjusted Kaplan–Meier estimates of survival functions between the two treatment groups were computed and the log-rank test was used to compare the equality of the survival curves between them. The absolute reduction in the probability of the incidence of MACE within the 5-year follow-up was computed from these survival functions.

Finally, we tested for any violation of the proportional hazard assumption of the Cox regression model, first by adding an interaction term of the predictor; secondly by log-minus-log survival curves; and thirdly by Schoenfeld residuals tests.

#### Subgroup analysis

We also performed a subgroup analysis to explore the individual statins (atorvastatin, simvastatin, rosuvastatin and pravastatin) identified in the database. The primary and secondary endpoints were determined in these individual statin groups compared to the non-statin user group.

In all the analyses, we computed the point estimates with 95% confidence intervals (CI) at the conventional statistical significance level of 0.05. All analyses were conducted using Stata Software, version 14.

## Results

### Patient characteristics, cases and total follow up

**Fig. 1:**
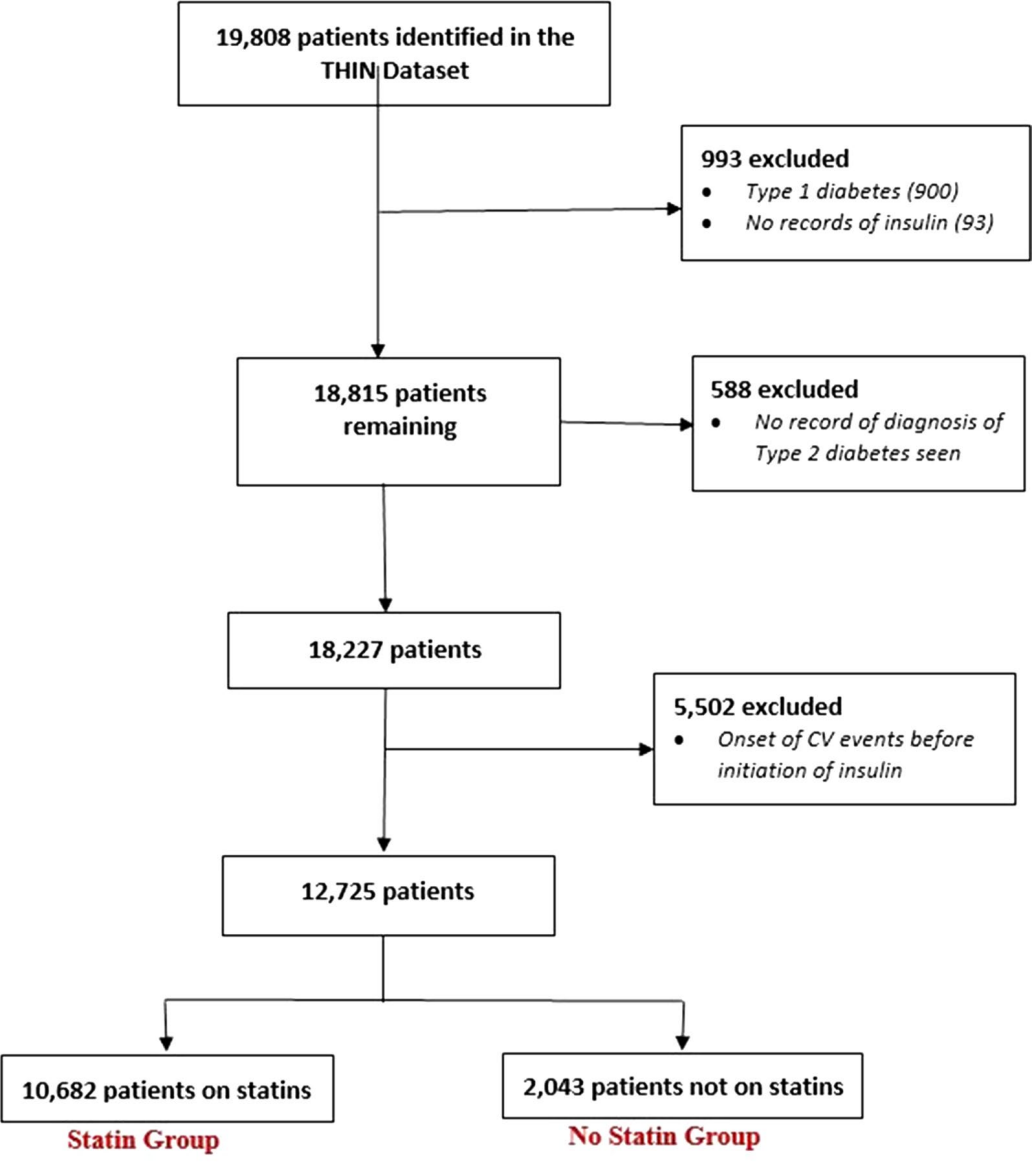
Selection of study participants

A total of 12,725 new insulin users with T2D (mean age 58.6 ± 14 years) were selected (Fig. 1). Among these 83.9% were statin users. Mean follow up was 3.9 ± 1.5 years, which represents a total follow-up of 49,516 person-years. Table 1 shows the baseline characteristics of the treatment groups. Non-users of statins were significantly younger, more likely to be female and had a significantly shorter duration of diabetes and insulin use. Baseline HbA1c, body weight and DBP were similar in the two groups (Table 1). SBP was higher in the statin users.

**Table 1:** Baseline characteristics of study participants

	Statin use	None	Total	p value
	(n = 10,682)	(n = 2043)	(n = 12,725)	
Demographics				
Age (years). mean (SD)	59.4 (12.7)	54.3 (18.2)	58.6 (13.8)	<0.001
Gender. no. (%)				
Female	5258 (49)	1122 (55)	6380 (50)	<0.001
Smoking status. no (%)				
Non-smoker	5549 (52)	1137 (56)	6686 (52)	
Ex-smoker	3634 (34)	543 (27)	4177 (33)	<0.001
Current	1499 (14)	363 (18)	1862 (15)	
Alcohol status. no (%)				
Never	3431 (32)	614 (30)	4045 (32)	
Ex-drinker	1133 (11)	320 (16)	1453 (11)	<0.001
Current	6118 (57)	1109 (54)	7227 (57)	
Deprivation. no (%)				
Least deprived	2151 (21)	403 (21)	2554 (20)	
Second quintile	2089 (20)	382 (20)	2471 (19)	
Third quintile	2255 (22)	422 (22)	2677 (21)	0.631
Fourth quintile	2171 (21)	399 (21)	2570 (20)	
Most deprived	1591 (16)	327 (17)	1918 (15)	
Clinical parameters. mean (SD)				
HbA1c (%)	8.7 (1.8)	8.7 (2.0)	8.7 (1.8)	0.556
BMI (kg/m^2^)	32.7 (6.9)	32.8 (7.2)	32.7 (6.9)	0.252
Diabetes duration^a^ (years)	4.0 (4.9)	2.5 (4.3)	3.8 (4.9)	<0.001
Time on insulin (years)	3.7 (6.3)	2.3 (6.0)	3.5 (6.3)	<0.001
Height (m)	1.7 (0.1)	1.7 (0.1)	1.7 (0.1)	0.860
Weight (kg)	91.4 (18.8)	91.9 (19.1)	91.5 (18.8)	0.268
DBP (mmHg)	76.5 (10.8)	76.9 (11.1)	76.6 (10.9)	0.129
SBP (mmHg)	136.4 (23.0)	134.6 (23.4)	136.1 (23.1)	0.002
Albumin (g/L)	4.1 (0.4)	4.0 (0.4)	4.1 (0.4)	0.002
ACR (mg/mol)	5.7 (8.4)	5.2 (8.5)	5.6 (8.5)	0.014
eGFR (mLs/min/1.73 m^2^)	64.6 (21.0)	68.0 (22.0)	65.1 (21.2)	<0.001
TC. (mmol/L)	4.6 (1.3)	4.7 (1.4)	4.6 (1.3)	<0.001
Triglyceride (mmol/L)	2.0 (1.2)	2.0 (1.2)	2.0 (1.2)	0.072
LDL (mmol/L)	2.4 (1.1)	2.5 (1.1)	2.4 (1.1)	<0.001
HDL (mmol/L)	1.3 (0.5)	1.3 (0.5)	1.3 (0.5)	0.225
BMI categories, no. (%)				
Normal	1371 (13)	294 (14)	1665 (13)	
Overweight	2533 (24)	489 (24)	3022 (24)	0.131
Obese	6778 (63)	1260 (62)	8038 (63)	
Use of medications, no. (%)				
Aspirin	10,481 (95)	1541 (94)	12,022 (94)	0.723
Antihypertensives	9321 (87)	1217 (91)	10,538 (83)	<0.001
GLTs^b^ no (%)				
Dual Therapy	2334 (22)	904 (44)	3238 (28)	
Triple Therapy	3400 (33)	609 (30)	4009 (33)	<0.001
More than triple therapy	4948 (45)	669 (26)	5478 (39)	
Comorbidities, no. (%)				
Hypoglycaemia	1824 (17)	313 (15)	2137 (17)	0.052
Heart failure	641 (6)	72 (4)	713 (6)	<0.001
PAD	756 (7)	67 (3)	823 (7)	<0.001
CHD	1390 (13)	75 (4)	1465 (12)	<0.001

*BMI* body mass index, *SBP* systolic blood pressure, *DBP* diastolic blood pressure, *HbA1c* haemoglobin A1c, *HDL* high-density lipoprotein, *LDL* low-density lipoprotein, *TC* total cholesterol, *GFR* glomerular filtration rate, *LLT* lipid lowering therapy, *PAD* peripheral arterial disease, *CHD* coronary heart disease, *ACR* albumin creatinine ratio, *SD* standard deviation

^a^Diabetes duration is time from first diagnosis of diabetes to date of insulin initiation

^b^GLTs (Glucose lowering therapies) denote the number of GLTs ever recorded for patients within the study duration and not the current regimen

### Primary endpoints: metabolic outcomes

#### Association between prior statin use and glycaemic response

**Fig. 2:**
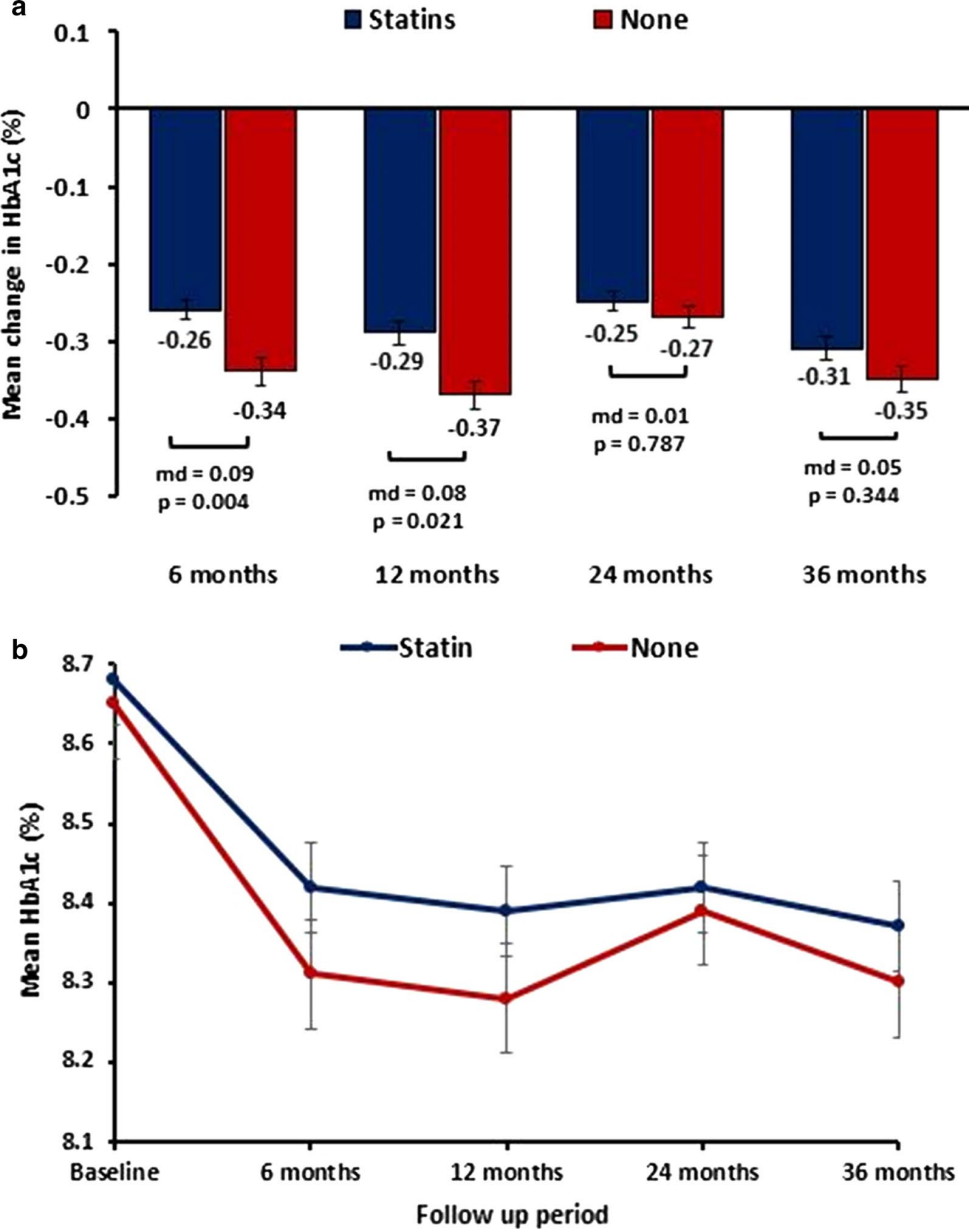
Mean differences in changes in HbA1c levels between the treatment groups (**a**) and the mean HbA1c levels in both treatment groups (**b**) (p < 0.05 from baseline for the study duration)

Glycaemic response was defined by the changes from baseline in HbA1c at 6, 12, 24 and 36 months after initiating insulin therapy. Figure 2a, b show the mean reductions in HbA1c and the mean HbA1c levels, respectively, at each of these time points. Both treatment groups (statin users and non-users) showed a significant reduction in HbA1c throughout the duration of the study (Fig. 2b) but insulin therapy resulted in a greater reduction in HbA1c among non-users of statins (Fig. 2a). This was significant at 6 months with mean reductions of −0.26% versus −0.34% (mean diff 0.09%; 95% CI 0.03–0.15; p = 0.004) in the statin user versus non-user groups respectively, but not at 36 months (corresponding mean reductions of −0.31% versus −0.35%; mean diff 0.05%; 95% CI −0.04 to 0.14; p = 0.172).

#### Relationship between statin use and other clinical measurements

There were increases in body weight following insulin initiation but no significant differences between the 2 groups from 6 to 24 months (Additional file 2). At 36 months, however, statin users showed a small reduction in weight of 0.18 kg (p = 0.031) versus an increase of 0.26 kg (p = 0.405) in the non-statin group (mean diff = −0.54 kg, 95% CI −0.35 to 0.88; p = 0.099).

SBP decreased in both treatment groups throughout the study, but more so in non-users of statins (Additional file 1) with a significant difference at 6 months (−1.27 vs −1.86 mmHg; p = 0.004) and 12 months (−1.5 vs −2.3 mmHg; p = 0.021). These differences were not maintained at 24 months (−1.6 vs −2.4 mmHg; p = 0.787) and 36 months (−2.4 vs −3.1 mmHg; p = 0.344). There was also a significant reduction in DBP in both treatment groups. Throughout the study, DBP was non-significantly lower among statin users compared to non-users. For example, the respective changes in DBP ranged from 0.74 versus 0.59 mmHg at 6 months to 2.0 versus 1.44 mmHg at 36 months (Additional file 1).

### Secondary endpoints: risk of major adverse cardiovascular events (3-point MACE)

#### Crude event rates

**Fig. 3:**
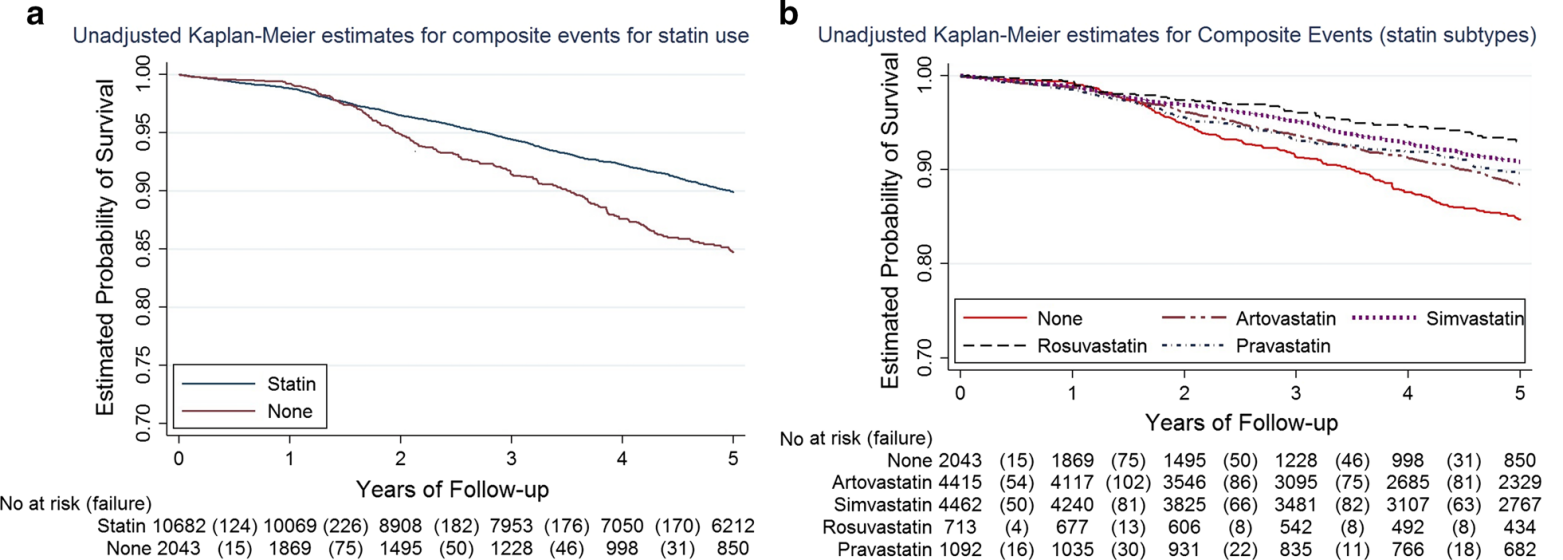
Kaplan-Meier curves showing the 5-year probability of survival for the 3-point composite MACE between the two treatment groups (**a**) and between the statin types (**b**). Log-rank test p < 0.0001 in both **a** and **b**

The probability of survival for the 3-point composite outcome of all-cause mortality, non-fatal MI and stroke was significantly different between the statin user versus non-user groups at 1-year (98.8% vs 99.2%) and 5-years (89.9% vs 84.7%) of follow-up (log-rank test p < 0.0001) (Fig. 3a). A total of 1095 (878 vs 217) composite events were reported with a crude event rate of 22.1 (20.7 vs 30.9) per 1000 person-years (95% CI 20.8–23.5) (Table 1). Table 2 shows a summary of the events for each of the individual MACE components with the absolute event rates.

**Table 2:** Events, crude incidence rates and hazard ratios of events in the two treatment groups

	Statin use (N = 10,682)	None (N = 2043)
Composite outcome^a^		
No of events/person-years	878/42,484	217/7032
Absolute rates^b^ (95% CI)	20.7 (19.3–22.1)	30.9 (27.0–35.3)
HR^c^ (95% CI)	1 (reference)	1.53 (1.32–1.77)
aHR^d^ (95% CI)	1 (reference)	1.36 (1.15–1.62)
All-cause mortality		
No of events/person-years	415/43,503	177/7098
Absolute rates (95% CI)	9.5 (8.7–10.5)	24.9 (21.5–28.9)
HR (95% CI)	1 (reference)	2.61 (2.19–3.12)
aHR (95% CI)	1 (reference)	1.89 (1.51–2.37)
Acute myocardial infarction (AMI)		
No of events/person-years	60/43,377	5/7094
Absolute rates (95% CI)	1.4 (1.1–1.8)	0.7 (0.3–1.7)
HR (95% CI)	1 (reference)	0.50 (0.20–1.25)
aHR (95% CI)	1 (reference)	0.88 (0.35–2.23)
Non-fatal stroke		
No of events/person-years	401/42,643	34/7051
Absolute rates (95% CI)	9.4 (8.5–10.4)	4.8 (3.4–6.7)
HR (95% CI)	1 (reference)	0.51 (0.36–0.72)
aHR (95% CI)	1 (reference)	0.55 (0.38–0.81)

^a^Composite outcome is a three-point MACE including all-cause mortality, non-fatal acute myocardial infarction (AMI) and non-fatal stroke

^b^Absolute rate at 1000 person-years

^c^HR (unadjusted hazard ratio)

^d^aHR (adjusted hazard ratio). Adjusted for age, gender, duration of insulin use, albumin, glomerular filtration rate, lipid profile, and coronary heart disease

#### Risk of 3-point composite of MACE

The risk of a MACE in the unadjusted model was 53% (aHR: 1.53; 95% CI 1.32–1.77) higher among non-users compared to statin users. This was reduced slightly to 36% (HR: 1.36; 95% CI 1.15–1.62) when we adjusted for age, gender, duration of insulin use, albumin, glomerular filtration rate, lipid profile, and coronary heart disease history. A similarly higher risk (89%) was observed for all-cause mortality (aHR: 1.89; 95% CI 1.51–2.37) among non-users of statins compared to statin users, but an opposite trend was observed for non-fatal MI and stroke: non-users of statins had a 12% lower risk of MI (aHR: 0.88, 95% CI 0.35–2.23) and a 45% lower risk of stroke (HR: 0.55; 95% CI 0.38–0.81) (Table 2).

### Subgroup and sensitivity analyses

Compared to non-users of statins, all of the individual statins showed higher HbA1c levels throughout the study (Additional file 2). The number of events for the 3-point composite of MACE was 398, 342, 41 and 461 among patients on atorvastatin, simvastatin, rosuvastatin and pravastatin respectively. The 5-year probability of survival differed significantly between the various statins (Fig. 3b; Log-rank test p < 0.0001). Table 3 shows a summary of these events, event rates and hazard ratios for the 3-point MACE composite and the individual components. When compared to non-statin users, the risk of the composite MACE was 18% lower (aHR: 0.82; 9% CI 0.68–0.98) among atorvastatin users, 33% lower (aHR: 0.67; 9% CI 0.55–0.82) among simvastatin users, 44% lower (aHR: 0.56; 9% CI 0.39–0.81) among rosuvastatin users and 22% lower (aHR: 0.78; 9% CI 0.60–1.01) among pravastatin users (see Additional file 2).

**Table 3:** Events, crude incidence rates and hazard ratios of events by statin types

	None	Atorvastatin	Simvastatin	Rosuvastatin	Pravastatin
	(N = 2043)	(N = 4415)	(N = 4462)	(N = 713)	(N = 1092)
Composite outcome^a^					
No of events/person-years	217/7032	398/16,840	342/18,287	41/2894	97/4461
Absolute rates^b^ (95% CI)	30.9 (27.0–35.3)	23.6 (21.4–26.1)	18.7 (16.8–20.8)	14.2 (10.4–19.2)	21.7 (17.8–26.5)
HR^c^ (95% CI)	1 (reference)	0.75 (0.64–0.89)	0.59 (0.50–0.70)	0.45 (0.32–0.62)	0.68 (0.54–0.87)
aHR^d^ (95% CI)	1 (reference)	0.82 (0.68–0.98)	0.67 (0.55–0.82)	0.56 (0.39–0.81)	0.78 (0.60–1.01)
All-cause mortality					
No of events/person-years	177/7098	203/17,229	162/18,710	17/2947	33/4616
Absolute rates (95% CI)	24.9 (21.5–28.9)	11.8 (10.3–13.5)	8.7 (7.4–10.1)	5.8 (3.6–9.3)	7.1 (5.1–10.1)
HR (95% CI)	1 (reference)	0.45 (0.37–0.55)	0.32 (0.26–0.40)	0.22 (0.13–0.36)	0.27 (0.19–0.39)
aHR (95% CI)	1 (reference)	0.61 (0.48–0.78)	0.49 (0.38–0.64)	0.39 (0.22–0.67)	0.39 (0.26–0.60)
Acute myocardial infarction (AMI)					
No of events/person-years	5/7094	26/17,168	21/18,670	2/2948	11/4590
Absolute rates (95% CI)	0.7 (0.3–1.7)	1.5 (1.0–2.2)	1.1 (0.7–1.7)	0.7 (0.2–2.7)	2.4 (1.3–4.3)
HR (95% CI)	1 (reference)	2.17 (0.83–5.65)	1.62 (0.61–4.30)	0.98 (0.19–5.03)	3.45 (1.20–9.94)
aHR (95% CI)	1 (reference)	1.42 (0.53–3.78)	0.88 (0.32–2.40)	0.63 (0.12–3.33)	1.61 (0.54–3.78)
Non-fatal stroke					
No of events/person-years	34/7051	168/16,919	158/18,337	22/2896	53/4489
Absolute rates (95% CI)	4.8 (3.4–6.7)	9.9 (8.5–11.6)	8.6 (7.4–10.1)	7.6 (5.0–11.5)	11.8 (9.0–15.5)
HR (95% CI)	1 (reference)	2.07 (1.43–2.99)	1.80 (1.24–2.61)	1.59 (0.93–2.72)	2.47 (1.61–3.80)
aHR (95% CI)	1 (reference)	1.82 (1.22–2.72)	1.67 (1.11–2.51)	1.64 (0.91–2.94)	2.42 (1.52–3.84)

^a^Composite outcome is a three-point MACE including all-cause mortality, non-fatal acute myocardial infarction (AMI) and non-fatal stroke

^b^Absolute rate at 1000 person-years

^c^HR (unadjusted hazard ratio)

^d^aHR (adjusted hazard ratio). Adjusted for age, gender, duration of insulin use, albumin, glomerular filtration rate, lipid profile, and coronary heart disease

Finally, to test the adequacy of our multiple imputation approach in addressing the impact of some missing data in our dataset, we conducted a sensitivity analysis wherein the primary endpoints in the imputed dataset were compared with the dataset with missing values and found to be similar, thereby affirming the robustness of the imputation method employed. The proportional hazards assumption was examined by comparing the cumulative hazard plots grouped on exposure; no violations were observed.

## Discussion

Although statins are well recognised to confer cardio-protective benefits [1–3], there is increasing evidence of a link between statin use and new-onset diabetes [6–8]. The cardiovascular benefits usually outweigh the risk of developing insulin resistance [22]. More recently, systematic reviews of randomised trials and observational studies have shown a modest adverse effect of statin therapy on glycaemic control (specifically HbA1c) [10–14]. In a meta-analysis of 9 randomised trials involving 9696 participants with a mean follow-up of 3.6 years, the average HbA1c of those randomised to statin treatment was 0.12% (95% CI 0.04, 0.20) higher compared with those randomised to placebo [15]. There was also some heterogeneity of effect among the different statins [15]. However, this small effect on HbA1c may be an underestimate of the true effect of statins on glycaemic control in everyday practice because of the selective nature of patients included in RCTs, who are likely to be more compliant and motivated, and there may be bias in the data due to an unbalanced need to adjust GLTs between the statin and control groups. Thus, caution is needed in the extrapolation of RCT-derived estimates of clinical outcomes when formulating guidelines for routine clinical practice. For ample, in a large observational cohort study the differences in HbA1c between statin users and non-users was much greater than that previously reported from RCTs [23].

### Clinical implication

Previous observational studies and RCTs have not reported the effects of concomitant statin use on HbA1c or CV outcomes among those patients who are often at highest risk with established T2D and who are newly started on insulin therapy [23, 24]. This is an important and growing sub-population as primary care clinicians are challenged to up-titrate medication, seek better control of diabetes symptoms and HbA1c, and to judge the overall risks and benefits, the alternatives and the optimal timing of commencement of insulin therapy. Given that insulin-initiated patients with T2D are likely to be more complex, have longer disease duration and a much higher risk of CV disease, the present study provides important new information about the effects of background statin therapy on metabolic and CV outcomes in the first 5 years after starting insulin.

Several conclusions can be derived from this large cohort study. Firstly, despite significant reductions in HbA1c in both groups following commencement of insulin therapy, non-users of statins achieved better HbA1c lowering compared with statin users. There were no differences in insulin-induced weight gain between the two groups but systolic BP was also lower in the non-statin users. It is notable in the present study that patients receiving statins were older, and had longer duration of diabetes and insulin treatment compared to non-users. However, the lower HbA1c reduction among statin users persisted even after adjusting for multiple confounders.

Secondly, compared with non-users, all of the individual statins were associated with worse HbA1c outcomes. This contrasts with previous RCTs which have suggested differential adverse effects on glycaemic control among the different statins [11–15]. However, in a real-world cohort study of insulin-treated patients, any differential effect of different statins on glycaemic control may have been offset by differences in insulin dose adjustment.

Despite achieving less of a reduction in HbA1c, statin users had a significantly higher probability of survival for the 3-point composite MACE outcome (all-cause mortality, non-fatal MI and stroke) but this finding was not replicated when considering the individual risks of non-fatal MI or stroke. In this regard, it is worth noting that from RCTs the reduction in CV events is linearly related to LDL-cholesterol (LDL-C) reduction, especially in the first year of treatment, but observational studies comparing different statins in routine clinical practice have reported inconsistent outcomes depending upon the endpoints examined and the methods used [25–27]. Thus, discordance between RCT and observational data on statins is not unusual, but in this study the overall ‘real life effect’ of statins on MACE and all-cause mortality reduction seems to be consistent with the established evidence from statin trials. However, in a similar real clinical setting, unlike our in our study, moderate-intensity statin treatment was found to be ineffective in cardiovascular primary prevention for patients with diabetic nephropathy [28]. This can be explained by the differences between these two populations, nonetheless, it provides a good insight as the mean eGFR our population was 66 mL s/min/1.73 m^2^.

### Possible underlying mechanisms

Several mechanisms have been proposed to explain how statins may worsen dysglycaemia: (1) impaired pancreatic β-cell secretion of insulin due to direct and indirect effects on calcium channels; (2) reduced insulin-mediated glucose uptake, especially in skeletal muscle, due to impaired expression and/or translocation of GLUT4 transporters from the cytosol to the plasma membrane; and (3) exacerbation of insulin resistance in muscle, liver and adipose tissues via multiple effects on insulin signal transduction, e.g. depletion of coenzyme Q10, inhibition of phosphorylation events downstream of the insulin receptor and inhibition of adipocyte differentiation [29]. In addition, statins have varying degrees of lipophilicity. The lipophilic statins diffuse passively through the hepatocellular membrane to inhibit HMG-CoA reductase in the liver but they can also diffuse passively and easily into extrahepatic tissues whereas the hydrophilic statins require carrier-mediated transport to enter cells. This may explain some of the observations of a higher incidence of new-onset diabetes with the lipophilic statins, e.g. simvastatin and atorvastatin, via effects on peripheral insulin sensitivity [30]. Similarly, a 6-year follow up study of the METSIM cohort showed that statin treatment increases the risk of type 2 diabetes by 46%, attributable to decreases in insulin sensitivity and insulin secretion [31], while simvastatin may induce insulin resistance through a novel fatty acid mediated cholesterol independent mechanism [32].

### Strength and limitations

Our analyses are subject to a number of limitations including allocation bias and residual confounders. This includes possible differences in compliance, indications for intensification of treatments, markers of β-cell deterioration, frequency of hypoglycaemia and some lifestyle and dietary factors which could influence our findings. Although we could not exclude the possibility of residual confounders, we were able to account for differences in the observed covariates and used robust analytical techniques to control for any confounding factors that may bias the results of the estimated treatment effects.

Our data were derived from a large UK database and therefore cannot be generalised to other ethnic groups. We were also unable to obtain longitudinal data on insulin doses, which are important in assessing insulin-induced weight gain [33], which in turn could potentially influence glycaemic and CV outcomes.

On the other hand, a real strength of this study is the relatively large number of patients, the availability of, and adjustment for, a large number of known confounders, and the routine systematic nature of data collection in THIN which reflects real world practice and outcomes. We have also focused this study on an important patient population, which has not been previously studied.

## Conclusions

In summary, in a large cohort of T2D patients who are newly started on insulin therapy, and who reflect a real-world population in routine clinical practice, we have confirmed the major benefits of statin use in reducing CV risk and mortality. We have also quantified, for the first time, the modest adverse effects of background statin use on the HbA1c outcomes following insulin initiation. It is clear that, in this high-risk population, the benefits of statins on CV outcomes outweigh the small adverse metabolic effects on glycaemic control.

## Additional files

**Additional file 1.** Mean change and differences in weight (A), Systolic Blood Pressure (B) and Diastolic Blood Pressure (C) between the two treatment groups. Mean changes in HbA1c in the different types of statins, compared to non-statin users (D). P value for all is <0.05.

**Additional file 2.** Events and hazard ratios of CV events between non-statin vs non-statin users and statin type.

## Abbreviations

CV: cardiovascular
DBP: diastolic blood pressure
HbA1c: glycated hemoglobin
MACE: major adverse cardiovascular event
RCT: randomised controlled trial
SBP: systolic blood pressure
T2D: type 2 diabetes
THIN: The Health Improvement Network
