# Supplementary figures and images for: Effects of background statin therapy on glycemic response and cardiovascular events following initiation of insulin therapy in type 2 diabetes: a large UK cohort study
Source: Cardiovasc Diabetol. 2017 Aug 22;16:107. doi: 10.1186/s12933-017-0587-6 (PMC5567903; doi:10.1186/s12933-017-0587-6)

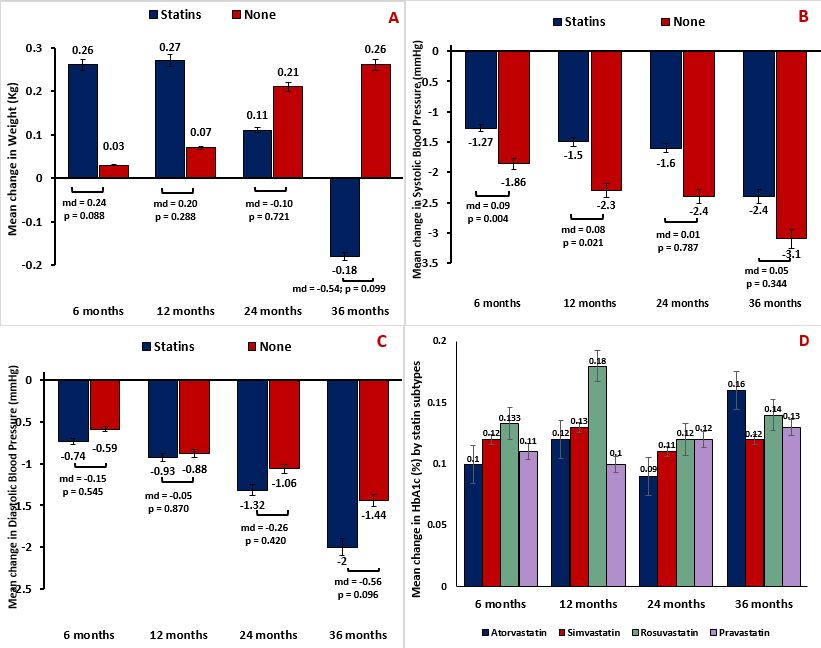

Supplement: Supplementary file 1 — Additional file 1. Mean change and differences in weight (A), Systolic Blood Pressure (B) and Diastolic Blood Pressure (C) between the two treatment groups. Mean changes in HbA1c in the different types of statins, compared to non-statin users (D). P value for all is <0.05. [file 12933_2017_587_MOESM1_ESM.tif]

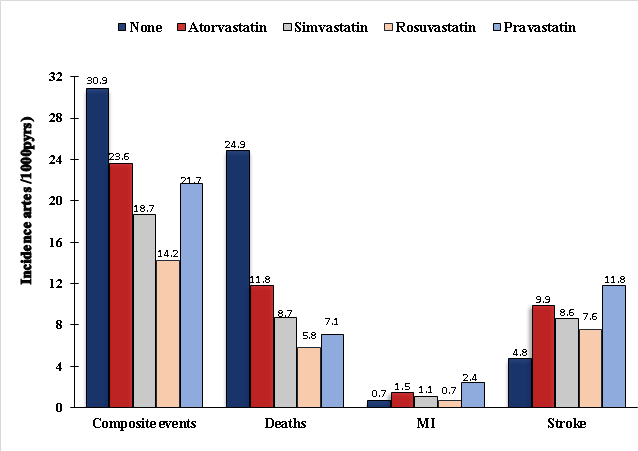

Supplement: Supplementary file 2 — Additional file 2. Events and hazard ratios of CV events between non-statin vs non-statin users and statin type. [file 12933_2017_587_MOESM2_ESM.zip › supplementary_1b.tif]

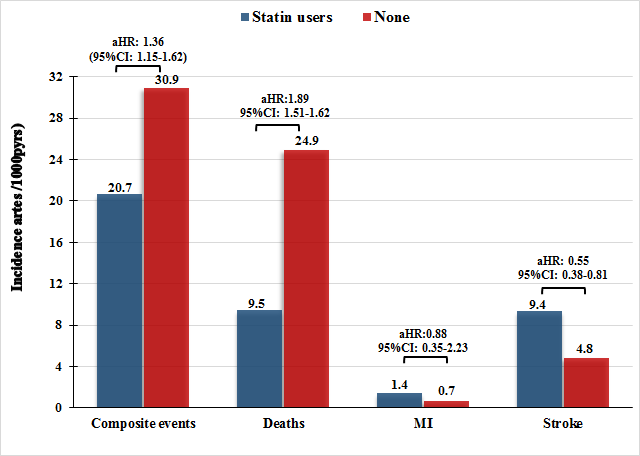

Supplement: Supplementary file 2 — Additional file 2. Events and hazard ratios of CV events between non-statin vs non-statin users and statin type. [file 12933_2017_587_MOESM2_ESM.zip › Supplementary_1a.tif]
